# Reliability of INTERMED Spanish version and applicability in liver transplant patients: a cross-sectional study

**DOI:** 10.1186/1472-6963-11-160

**Published:** 2011-07-05

**Authors:** Elena Lobo, MªJosé Rabanaque, MªLuisa Bellido, Antonio Lobo

**Affiliations:** 1 Departamento de Medicina Preventiva y Salud Pública, Universidad de Zaragoza, Domingo Miral s/n, 50009 Zaragoza, Spain; 2 CIBERSAM, Grupo 13, Instituto de Salud Carlos III, Spain; 3 Servicio de Psiquiatría, Hospital Clínico Universitario, S. Juan Bosco, 15. 50009 Zaragoza, Spain; 4 Departamento de Medicina Psiquiatría y Dermatología, Universidad de Zaragoza, Domingo Miral s/n, 50009 Zaragoza, Spain

## Abstract

**Background:**

Integrated delivery methods in healthcare systems have been proposed to confront the increasing complexity in general health care. INTERMED is an empirically derived, observer-rated instrument to measure case complexity. It was intended as a visualized, action-oriented decision-support tool for the early assessment of bio-psychosocial health risks and health needs. This study aims to document the reliability and applicability of the Spanish version of INTERMED in liver transplant patients.

**Methods:**

Cross-sectional study of patients who had been included in the liver transplant waiting list. Two nurses interviewed the patients with INTERMED, and scored the instrument blind to each other. Kappa and w-kappa, Spearman, Kendall and intraclass correlation coefficients, and Cronbach's alfa were calculated.

**Results:**

No patient refused the interview. Satisfactory coefficients were documented in most INTERMED items. Kappa was = 0.858 for the categorization of patients as "complex", and 21 of them (48.8%) were classified in this category, and were considered to need integrated treatment.

**Conclusions:**

The Spanish version of INTERMED is reliable. Its applicability in liver transplant patients adds to its generalizability.

## Background

Complexity in general health care is increasingly prevalent because of the growing number of patients who have multi-morbid conditions, and the increased technical possibilities of medicine [1]. Furthermore, social problems [2], and particularly behavioural health disorders, which may affect 40% or more of medical inpatients, interfere with medical care adding up to this complexity [3]. Social problems and mental health disorders are usually managed independently from general somatic disorders, and this care fragmentation has been shown to result in medical and behavioural disorders more persistent with increased complications and greater disability, leading to higher total health care and disability costs [3].

Integrated delivery methods in healthcare systems have been proposed to confront the complex problems found in present day medicine [4]. In the context of theoretic approaches to case complexity in the medically ill [1], a new instrument was empirically derived, INTERMED [5], its conceptual model being based on observations that comprehensive understanding of patients' needs increases effectiveness of healthcare delivery [6], The concept measured is case complexity [7-9]. Specifically, INTERMED classifies information into four domains (biological, psychological, social and healthcare), and was intended to universally screen at admission to medical wards, for the early identification of patients with multiple care needs. The utility of this method has been shown in different cultures [10] and in different types of both ambulatory populations and in-patients [11,12], including elderly patients [13]. INTERMED provides a quick overview of the patient's vulnerabilities [14], so that an integrated treatment plan can be formulated to counteract fragmentation of medical care [3]. It also provides a model to communicate these potential problems to the medical staff and to facilitate an early referral [15].

The reliability of INTERMED, which may be applied by nurses, was reported in a trauma center [7] and then in a heterogeneous sample of patients with somatic conditions [16]. The validity of this instrument has been documented in patients admitted to internal medicine [9], and also in specific diagnostic groups including patients in dialysis [17], with low back pain [18], terminal cancer [19], diabetes [2], rheumatoid arthritis [20], multiple sclerosis [11] and pulmonary patients [21]. It has also been shown that compared with usual care, nurse-led interventions based on INTERMED scores resulted in improvements in quality of life at discharge in a sample of general medical patients [22].

However, the applicability of INTERMED in transplant patients has not been reported until now. The early evaluation of health risks may be essential for optimal care in such patients, since complexity is anticipated in view of common medical co-morbidities and complications of medical and surgical procedures, and the fact that they often require a life-long regimen of immunosuppressive medication and dependency on the transplant team [23]. The burden of psychosocial problems in transplant patients has been considered to be associated with potential morbidity and mortality [24], and the improvement of the accuracy and meaningfulness of assessment, by incorporating biological, psychological, and contextual factors that may contribute to negative adjustment has been emphasized [25].

A Spanish version of INTERMED has been developed [21]. However, the reliability of this version has not been adequately documented. This is the first report intended to document the applicability of INTERMED in transplant patients, and the first one to document the reliability of the Spanish version.

## Methods

### Setting, study design and sample

Patients who had been included in the liver transplant waiting list of the Hospital Clinico Universitario in Zaragoza, Spain were invited to participate by a research nurse.

The study participants were interviewed by this research nurse and a consultation-liaison nurse, alternating the position of interviewer and observer. Following the interview the patients were scored by both nurses, blind to each other and using the INTERMED anchor points. Once the scores were computed, a between-rater consensus score for each INTERMED item was decided, to be used in some statistical analysis.

### Instrument

INTERMED is based on a review of the medical chart and a patient semi-structured interview, which is designed like a medical anamnesis but complemented by psychosocial and health care-related information. It classifies the data into the four domains related to biopsychosocial and health care aspects of disease, and each domain has five variables, related to "history," "current state," and "prognoses". The twenty resulting variables are rated 0 to 3 [15] according to a manual with clinical anchor points, the potential score ranging 0 to 60, obtained by adding-up the individual variables, indicating the patient's level of care needs [5,7].

The instrument was previously translated into Spanish by an experienced consultation-liaison psychiatrist along with the research nurse, and went through the forward and backward translation process. Then, in a pilot study among transplant patients in the same hospital, 19 patients were interviewed with INTERMED by two trained nurses in a procedure similar to the one used in this study. The method was considered to be feasible and acceptable to patients, physicians and nurses. Inter-rater agreement coefficients were in general satisfactory (kappa >0.6), but refining the training was recommended, as it has been done for this study [26].

For the present report patients were classified by INTERMED as "complex" and "non-complex" on the basis of the optimal cut-off score (20/21) for the need of integrated treatment documented in previous studies [27,21].

### Statistical analysis

Inter-rater reliability was calculated for individual items in INTERMED, as well as for the categorization of patients as "complex" or "non-complex". Both, kappa and weighted-kappa (w-kappa) coefficients were used. To further explore reliability, both Spearman and intraclass correlation coefficients between the raters were calculated for the INTERMED total score and the four domain scores. Kendall's coefficient was calculated for the categorical variable, namely the distinction between "complex" and "non-complex" patients. The consensus score for each INTERMED item was used for calculating the internal consistency of the instrument, and Cronbach's alfa was applied for these calculations. As in the original report, we further studied the structure of INTERMED by calculating Spearman correlation coefficients among domain scores [27]. Data were analyzed using Epidat and SPSS14.0 software.

This study was approved by the Ethical Committee Research Comission of the Hospital Clinico Universitario.

## Results

Forty three patients were assessed with INTERMED (mean age 52.4 ± 8.7; female sex 30%). No patient refused the interview, which was completed in an average of 20 minutes per patient.

Table 1 shows inter-rater reliability coefficients for each INTERMED item. Satisfactory agreement was observed in most items. Kappa values below 0.5 were only observed in psychological and social prognoses, and w-kappa values were better in almost all items. Satisfactory agreement was also observed in the categorization of patients as "complex" or "non-complex" in INTERMED (kappa = 0.858).

**Table 1:** Inter-rater reliability coefficients, by INTERMED item

DOMAINS	ITEMS	Kappa	Kappa Weighted*
	Chronicity	1	1
	
	Diagnostic dilemma	0.76	0.87
	
**BIOLOGICAL**	Severity of symptoms	0.69	0.73
	
	Diagnostic challenge	0.65	0.89
	
	Prognosis - Complications life threat	0.86	0.87

	Restrictions in coping	0.75	0.93
	
	Psychiatric dysfunctioning	0.69	0.85
	
**PSYCHOLOGICAL**	Resistance to treatment	0.61	0.69
	
	Psychiatric symptoms	0.75	0.84
	
	Prognosis - Mental health threat	0.42	0.31

	Restrictions in integration	0.62	0.78
	
	Social dysfunctioning	0.75	0.80
	
**SOCIAL**	Residential instability	0.57	0.69
	
	Restrictions of network	0.63	0.66
	
	Prognosis - Social vulnerability	0.48	0.56

	Intensity of treatment	0.71	0.69
	
	Treatment experience	0.79	0.84
	
**HEALTH CARE**	Organisation of care	0.83	0.91
	
	Appropriateness of referral	1	1
	
	Prognosis - Coordination	0.52	0.74

*Kappa weighted with quadratic wheights.

Table 2 shows the mean scores by domain and mean total scores, which were similar in both raters. Means of the consensus scores were: biological = 7.64 (± 1.49); psychological = 4.63 (± 2.41); social = 1.95 (± 1.86); health care = 6.67 (± 1.86). Between-rater correlation coefficients for the INTERMED total score and for the four domain scores were also quite acceptable (table 2). For the distinction between "complex" and "non-complex" patients Kendall's coefficient was 0.867. Cronbach's alfa coefficient for INTERMED was 0.71. Between-domain correlations are shown in table 3. Positive correlations are observed in general, the exception being correlations with the social domain.

**Table 2:** Mean scores by INTERMED domain, mean total scores, and between-rater correlation coefficients

Domain	Rater 1				Rater 2				Spearman	Intraclass
	**Mean**	**Min**	**Max**	**SD**	**Mean**	**Min**	**Max**	**SD**		

BIOLOGICAL^♦^	7.7	6	12	1.3	7.7	6	12	1.5	0.91*	0.92*

PSYCHOLOGICAL^♦^	4.2	0	9	2.3	4.6	1	9	2.2	0.87*	0.87*

SOCIAL^♦^	1.4	0	6	1.6	2.0	0	8	2.1	0.91*	0.76*

HEALTH CARE^♦^	6.6	3	10	1.8	6.3	3	11	1.7	0.91*	0.89*

INTERMED Score^♦♦^	19.9	13	30	4.4	20.5	12	32	4.8	0.92*	0.91*

^♦^Range 0-15; ^♦♦^Range 0-60; *p < 0.001

**Table 3:** Spearman correlations among INTERMED domain scores

	BIOLOGICAL	PSYCHOLOGICAL	SOCIAL
	**Spearman**	**Spearman**	**Spearman**

PSYCHOLOGICAL	0.32*	-	-

SOCIAL	0.05	0.08	-

HEALTH CARE	0.28	0.38*	0.24

*p < 0.05

In relation to applicability of INTERMED in liver transplant patients, 21 of them (48.8%) scored above the cut-off score (20/21). The highest scores were observed in the biological and health care domains, but high scores were also given to the psychological domain (table 2). Five (11.6%) patients scored more than one standard deviation above the mean in the biological domain; eight (18.6%) patients in the psychopathological domain; ten (23.2%) patients in the social problems domain; and the same proportion in the healthcare domain.

## Discussion

One objective of this paper was to assess the reliability of the Spanish version of INTERMED, and the overall results were satisfactory. The raters' agreement in the categorization of patients as "complex" or "non-complex" was high (k = 0.858). Similarly, inter-rater kappa (k) coefficients were also satisfactory for most INTERMED individual items. The measurement of agreement is difficult, particularly when the variables of interest have more than two possible values. However, there is widespread consensus regarding the advantages of the stringent kappa (k) coefficient, which takes into account chance agreement. The main weakness of k is that it measures the frequency of exact agreement, rather than the degree of approximate agreement. However, to take this into account we have also calculated weighted kappa in this particular study, the agreement coefficients being even better. Furthermore, the reliability of this Spanish version of INTERMED was also supported by satisfactory Spearman and intraclass correlation coefficients, when the measures were close to a continuous distribution, namely, INTERMED total scores and individual domain scores.

In one of the original studies, DeJonge et al reported Spearman coefficients ranking from 0.91 to 0.96 in assessing the different domains, and considered "very good" the agreement in the categorization of patients as "complex" or "non-complex, the only variable they assessed with kappa (k = 0.85) [16]. Therefore, this study on the Spanish version of INTERMED is in agreement with and supports the results reported with the original version.

The original authors have suggested that inexperienced raters may need more training. Consequently, the satisfactory results with the Spanish version of INTERMED are remarkable, since one of the raters was a research nurse without clinical experience. Moreover, the agreement of experienced and inexperienced raters supports the "procedural validity" of INTERMED. Procedural validity speaks only of the evaluation procedure and not to the validity of the construct itself, and is defined as the extent to which a new diagnostic procedure yields results similar to the results of an established diagnostic procedure that is used as a criterion [28]. In the absence of an established "gold standard," the experienced clinicians' judgement has been used as a comparative criterion when researchers with little clinical experience are trying to use a new diagnostic instrument [28,29]. It might be argued that further training and/or refinement of items related to psychosocial prognosis should be considered, since k values obtained were below 0.5. In fact, in the first reliability study by Huyse et al inconsistent results were reported for these items, the agreement coefficients being low in some (ICC were 0.49 and 0.84 for the psychological prognosis; and 0.80 and 0.42 for the social prognosis; Kendall's coefficients were 0.52 and 0.36 for the psychological prognosis and 0.25 and 0.70 for the social prognosis) [7]. However, that might be the closest one can come with a prognostic item, since prognosis in medicine is always difficult (with regard to any outcome) in view of the uncertain future.

Internal consistency of the Spanish INTERMED may also be considered to be acceptable (alfa = 0.71). Slightly higher coefficients have been reported with the original version of INTERMED (alfa >0.78) [27]. However, as suggested by De Jonge et al, values higher than 0.8 can not be demanded, not even desired, in view of the heterogeneity of domains included in INTERMED [16]. In support of this view, between-domain correlations were statistically significant, but modest (Sperman's around 0.3), and the correlations with the social domain were non-significant. In fact, common clinical sense does not require in liver transplant patients that, for example, the severity of the biological condition should have a proportional counterpart of social problems.

In relation to applicability, the second objective in this report, no patient refused the interview. It is noticeable that nurses, even with limited clinical experience in patient care were able to detect in a 20 minutes interview, early after inclusion of patients in the transplant waiting list, that almost half of them had high levels of potential complexity. "Complex" patients in INTERMED have been considered to need integrated treatment [30]. The needs of psycho-social attention in this particular sample are supported by the proportion of patients scoring above the 20/21 threshold point proposed (48.8%). Previous studies have also shown considerable proportions of complex patients according to the same INTERMED criteria: 62.3% among diabetic patients [2] and 12.8% or 21.4 (%) among multiple sclerosis patients [11]. Physicians are well prepared to detect the severity of the medical condition, but under-detection of psychopathological and social problems is common in medicine and has been associated with negative outcomes [3]. Specifically, psychopathological problems in liver transplant patients have been considered to be associated with poor prognosis [24]. Therefore, the early evaluation and then the appropriate management of complex transplant recipients might be essential for optimal patient care [23].

Fragmentation of care into biological, psychopathoplogical and social care may be even more detrimental in liver transplant patients than in other, less complex medical patients [3]. The early detection with an instrument such as INTERMED, intended to enhance the communication between patients and the health providers as well as between different professions and disciplines [31] should facilitate the early development of individualized, integral care planning. Coordination between professionals, which may also be facilitated by the use of INTERMED, has been suggested to affect patients' clinical outcomes and satisfaction with their care [32,33]. Efforts have been done to improve health service utilization and costs with new models of guided, comprehensive healthcare provided for people with complex health conditions [34,35]. There is some evidence of improved health outcomes following an intervention targeted for complex medical patients identified by INTERMED [36].

In support of the applicability of the use of INTERMED, this study was completed in a socio-cultural environment different from most previous studies. Patients in this sample were recruited in a rather typical public hospital in Spain, which covers a geographical health area and populations with a wide range of socio-economical background. Therefore, the results support its future use in Spanish speaking populations.

## Conclusions

The Spanish version of INTERMED is reliable. Moreover, since the validity of this instrument was previously documented in different types of medical patients, this study adds to the applicability of INTERMED in special populations such as the liver transplant patients, with high levels of complexity as shown in this study. The usefulness of INTERMED in studies such as the ongoing multi-national, European study in liver transplant patients [37] is now further supported. However, its utility in improving health care delivery for complex liver transplant still needs to be evaluated.

## Competing interests

The authors declare that they have no competing interests.

## Authors' contributions

EL participated in the design, carried out the study, interviewed the patients, prepared the dataset, analysed and prepared all the results and drafted the manuscript. MJR participated in the design of the study and revised critically the manuscript. MLB participated in the design of the study, carried out interviews with patients and helped draft the manuscript. AL conceived the study and designed it, helped to draft the manuscript and made important final modifications. All authors read and approved the final manuscript.

## Pre-publication history

The pre-publication history for this paper can be accessed here:

http://www.biomedcentral.com/1472-6963/11/160/prepub
